# Enhancing spoof surface-plasmons with gradient metasurfaces

**DOI:** 10.1038/srep08772

**Published:** 2015-03-05

**Authors:** Ling-Bao Kong, Cheng-Ping Huang, Chao-Hai Du, Pu-Kun Liu, Xiao-Gang Yin

**Affiliations:** 1 School of Electronics Engineering and Computer Science, Peking University, Beijing 100871, P.R. China; 2 Department of Applied Physics, Nanjing Tech University, Nanjing 210009, P.R. China; 3 College of Science, Nanjing University of Aeronautics and Astronautics, Nanjing 210016, P.R. China

## Abstract

The coupling between surface plasmons and free electrons may be used to amplify waves or accelerate particles. Nonetheless, such an interaction is usually weak due to the small interaction length or velocity mismatching. Here a mechanism for enhancing the coupling between plasmonic fields and relativistic electrons is proposed. By using a weakly gradient meta-surface that supports the spoof surface-plasmons (SSP), the phase velocity of SSP mode can be manipulated and quasi-velocity-matching between SSP and electrons may be achieved. The dynamic coupling equations suggest that, due to the strong coupling, the energy can be extracted continuously from the relativistic electrons. The sustained increase of SSP in a narrow frequency band has been demonstrated by the particle-in-cell simulations, where the output power of SSP attains 65 W at 1 THz (with 28 mm interaction length) and the coupling efficiency is enhanced by two orders of magnitude. The results may find potential applications for designing new compact and efficient THz wave sources.

In vacuum electronics, the interaction between the electromagnetic fields and charged particles plays a crucial role. Such an interaction can be used to amplify the electromagnetic waves or accelerate the charged particles^1^,^2^,^3^. A well-known example is the electron cyclotron maser, which acts as an important coherent high-power microwave source. In solid-state physics, the interaction between the photons and electrons is also responsible for many interesting physical properties^4^. For example, due to the coupling of light with free electrons in a metal, the surface- plasmon polariton (SPP) mode can be induced on the metal surface, leading to subwavelength plasmonic waveguiding and enhanced optical transmission etc.^5^,^6^,^7^. In some cases, the studies of the vacuum electronics and plasmonics could be combined together. It was shown that, when an electron beam passes through an unstructured metal film (or near a metallic nanostructure), the propagating SPP mode (or localized plasmon mode) can be excited^8^,^9^. Moreover, based on this effect, a Cherenkov radiation source operating in the visible or ultraviolet frequency range was proposed^10^. Recently, with the structured metal surface, the generation of spoof surface-plasmons (SSP) with free electron bunches has also been studied^11^,^12^, yielding an effective surface mode mimicking the SPP waves.

An efficient energy transfer between the electron and SPP requires that the electron can be accelerated or decelerated efficiently by the electric field. Although the SPP mode owns a strong field enhancement, the electron-wave interaction is usually less efficient. This is mainly due to the following reasons. Firstly, in the surface-normal direction, the strong normal component of the electric field will decay rapidly from the surface^13^,^14^, thus only allowing a small interaction length. Secondly, in the tangent or propagation direction, the electric field of SPP mode is very weak, especially in the low frequency such as the THz or microwave regime. Thirdly and most importantly, the electric-field force will change the velocity of electrons, especially in the strong coupling regime (this effect may be neglected in the weak coupling^10^,^11^,^12^). Thus, a significant velocity mismatch between the electrons and SPP waves (or relative motion of electrons with respect to the SPP field) can be resulted. Consequently, the electron travels in the accelerating and decelerating phases alternately, leading to the oscillation of efficiency with very small amplitude. These factors may pose a serious obstacle to the future study and applications.

Here we suggest that, by using the THz SSP on the metasurface, a *strong coupling* between the plasmonic fields and relativistic electrons can be realized. The key is that the effective index or phase velocity of SSP wave can be manipulated spatially with the weakly gradient metasurface, thus achieving a quasi-velocity-matching (QVM) between SSP and electrons. The dynamic coupling equations indicate that, with the QVM design, the energy of relativistic electrons can be extracted continuously and efficiently in short interaction length. We also present the particle-in-cell (PIC) simulations in the THz band, showing that the output power or coupling efficiency can be enhanced by two orders of magnitude.

## Results

**Figure 1:**
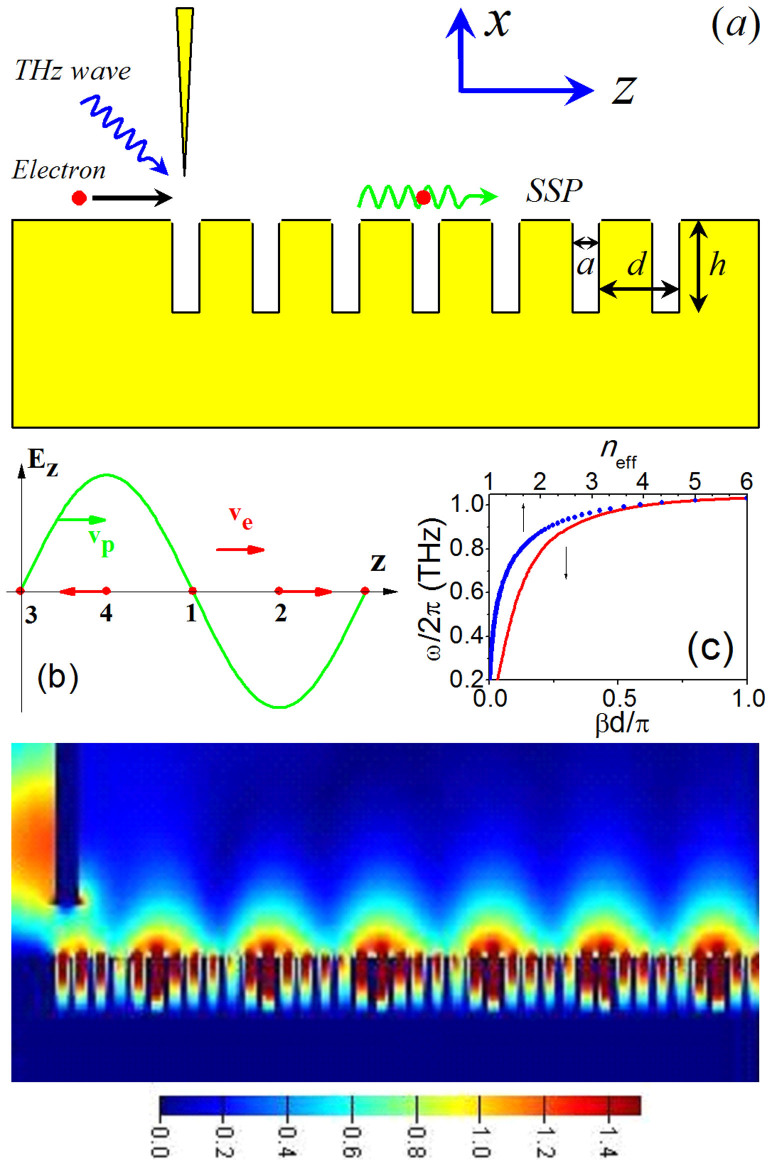
(a) Schematic view of the coupled system. (b) A tangential force is exerted on the electron: the electron can be accelerated or decelerated by the SSP. (c) The dispersion relation and effective index for the SSP. (d) The electric field (|Ez|) distribution of SSP mode (*d* = 24 *μm*, *a* = 15 *μm*, and *h* = 66 *μm*).

Metasurface may refer to a special kind of quasi-two-dimensional film surfaces, which are structured artificially with periodic, quasi-periodic or aperiodic order, thus owning exotic optical properties in such as the refraction, absorption, phase, and polarization, etc. Here, the metasurface under study is a metal surface milled with the periodic subwavelength grooves (the background material is assumed to be air or vacuum). The schematic view of structure is shown in Fig. 1(a), where the lattice constant is *d*, the groove width is *a*, and the depth of grooves is *h*. The relativistic electron with an initial velocity v_0_ (parallel to the metal surface; the initial distance between the electron and the surface is *x_0_*) is injected into the system. To localize the injected electron, a static magnetic field *B_0_* is applied along the z-axis. In the THz band, the metal behaves like a perfect electric conductor (PEC) and the true SPP mode will not work. However, the subwavelength corrugated metal surface can support an electromagnetic surface mode, i.e., the SSP mode^15^,^16^. The SSP mode can be induced externally by the edge coupling or self-excited by the electrons^11^,^17^. The excited SSP mode will interact with the relativistic electron via the tangent electric field (see Fig. 1b).

### Dynamics of relativistic electrons in the SSP fields

The dispersion of SSP mode can be deduced by analyzing the poles of reflection coefficients^16^. In the PEC approximation, the dispersion is obtained as:
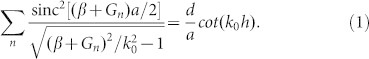
Here, *β* is the propagation constant of SSP mode, *G_n_* = 2*πn*/*d* (*n* = 0, ±1, ±2, ...) is the reciprocal vector, and *k*_0_ = *ω*/*c* is the wave vector in free space. In the long- wavelength limit, one can neglect the high-order modes and Eq. (1) may be simplified as 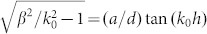
16. The dispersion and effective index of SSP mode (*n_eff_* = *β*/*k*_0_) are dependent on the structural parameters such as the groove width *a* or depth *h*, which provides the degrees of freedom for controlling the motion of SSP wave. Here we consider a structure with subwavelength dimensions: d = 24 μm, a = 15 μm, and h = 66 μm. Based on Eq. (1), the SSP dispersion and effective index were calculated and shown in Fig. 1(c). The effective index is larger than unity, indicating the slow-wave and bounded-mode characteristic. As an example, the electric-field distribution (|*E_z_*|) for 0.7 THz was simulated with the finite-difference time-domain (FDTD) method^18^, and the result is shown in Fig. 1d.

To study the dynamics of electron in the SSP wave, the fields excited above the corrugated metal surface can be expressed as 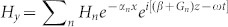 (here 
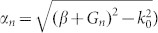
), which include multiple diffraction orders associated with the periodic structure. For the deep subwavelength structure studied here (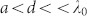), the corrugated metal surface behaves like an effective medium and the high-order modes can be neglected (See the supplementary materials). Consequently, the electromagnetic fields of SSP mode are written as (*x* ≥ 0)
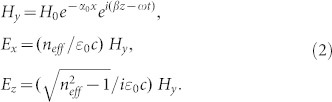
Here, *H*_0_ is the amplitude of SSP mode, which is determined by the intensity of excitation source. Equations (2) suggest that the electric field of SSP mode is governed by the effective index *n_eff_*. As *n_eff_* is obviously larger than unity, the tangent electric field will be comparable to the normal component (
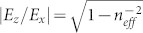
). In contrast, the tangent field of true SPP mode is usually much smaller than the normal one (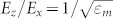, where *ε_m_* is the metal permittivity with 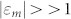
19). The enhanced tangent electric-field near the metal surface can also be seen clearly from Fig. 1(d). The enhanced tangent field will play a crucial role in the coupling.

The interaction between single relativistic electron and SSP mode is dominated by the Newton and Lorentz force equations:
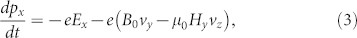

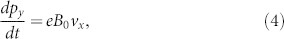

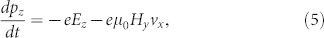
Here, *v_j_* (*j = x, y, z*) is the velocity of electron in the x, y, and z direction; −*e* is the charge of electron; *p_j_* = *γm*_0_*v_j_* is the relativistic electron momentum, where 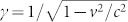 is the relativistic factor and *m_0_* is the rest mass of the electron. The energy transfer efficiency can be calculated with the loss of relativistic electron energy *η* = (*γ*_0_ − *γ*)/(*γ*_0_ − 1), where *γ*_0_ is the initial relativistic factor of electron.

### Coupling between SSP and electrons in a *homogenous* metasurface

**Figure 2:**
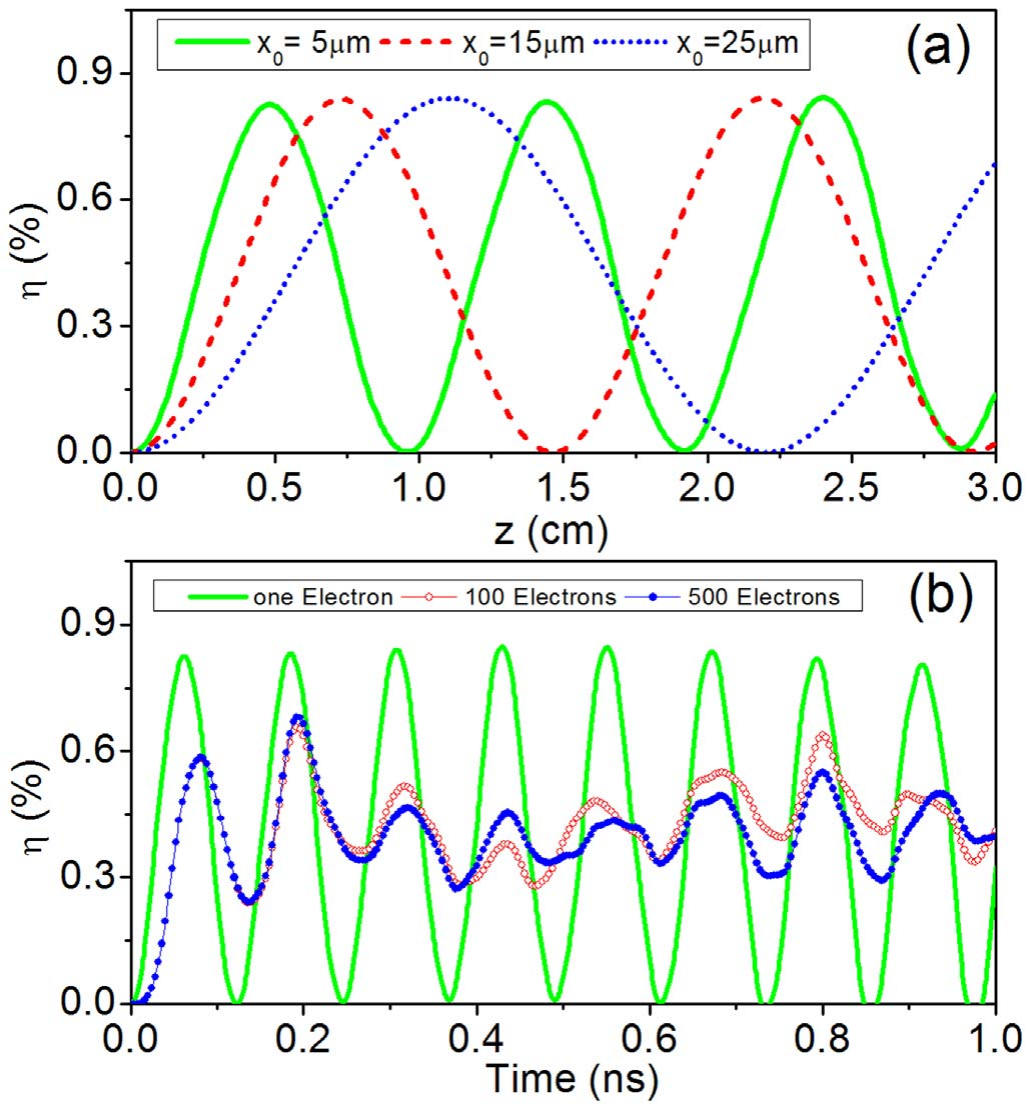
Coupling effect in the plasmonic system with the homogeneous metasurface. (a) The interaction efficiency is plotted as a function of length z, where x_0_ = 5, 15, and 25 μm, respectively. (b) The efficiency of one electron and average efficiency of 100 and 500 electrons (x_0_ = 5 μm). The input parameters are set as *v*_0_ = 1.002*v_p_*, *B*_0_ = 1*T*, and the SSP mode is externally excited with H_0_ = 0.2 A/mm.

Figure 2(a) shows the calculated efficiency *η* of the coupling, where the externally-excited SSP mode interacts with the electron along a homogeneous metasurface (the structure sizes are the same as that of Fig. 1c). Here, the working frequency is set as *f* = 1 THz (corresponding to an effective index *n_eff_* ≈ 3.835), the amplitude of SSP is H_0_ = 0.2 A/mm, and a static magnetic field *B*_0_ = 1 *T* is applied along the z axis. In contrast to the common belief where *v_e_* = *v_p_*^10^,^12^, our analysis suggests that an initial electron velocity close to but slightly larger than the phase velocity of SSP mode (*v_p_* = *c*/*n_eff_*) is more preferable for the coupling (See the supplementary materials). Thus we assume that the electron locates initially at point 3 of Fig. 1b with the velocity *v_0_* = 1.002*v_p_* in the z direction (the role of initial phase of SSP is discussed in the supplementary materials). Figure 2(a) demonstrates that the efficiency *η* is a periodic function of the interaction length. For different distance between the electron and metal surface (*x*_0_ = 5, 15, 25 *μm*), the coupling efficiencies are all close to 0.8% with the first saturated lengths of 0.48, 0.73, and 1.09 cm, respectively. The shorter saturated length for the smaller *x_0_* is due to the stronger SSP field near the metal surface. This character is advantageous for the design of a compact THz wave amplification device. The electron also presents a small oscillation along the x-axis (for *x*_0_ = 5 *μm*, 1.78 *μm* ≤ *x* ≤ 5.80 *μm*), which will not impinge on the metal surface (See the supplementary materials).

Figure 2(b) shows the comparison between the efficiency of one electron and the average efficiencies of 100 and 500 electrons (the electrons locate initially in one- wavelength length of the SSP wave with the equal electron-electron distance; *x*_0_ = 5 *μm*). The latter can mimic the behavior of quasi-continuous injection of electrons. The numerical studies show that the average efficiency of quasi-continuous electrons also oscillates with the time or length, with the oscillation periodicity being close to that of a single electron. Nonetheless, the coupling efficiency and oscillating amplitude are reduced slightly. The results suggest that the energy transfer can occur for beam electrons and that the single-electron method may elucidate the basic physics of the system.

### Coupling between SSP and electrons in a *gradient* metasurface

It is interesting to ask if there is any possible to extract the energy of relativistic electron more efficiently. The efficiency oscillation stems from the velocity mismatch between the electron and SSP mode (or the relative motion of electron with respect to SSP), as the relativistic electron will be substantially decelerated (at the wave crest) or accelerated (at the wave trough) by the strong SSP field. If we can slow down the SSP mode simultaneously and thus compensate the velocity mismatch completely or partly, a continuous deceleration of electron could be achieved. To realize such effect, the effective index of SSP mode needs to increase with the length gradually. Fortunately, the gradient metasurfaces provide the unique abilities for manipulating the motion of electromagnetic waves^12^,^19^,^20^,^21^,^22^. Here, we employ the subwavelength metallic grooves with the linearly varying groove width:
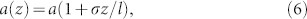
where *σ* is a small positive parameter denoting the weak change of groove width, *l* is the length of metasurface. For metallic grooves with the increasing width along the z-axis, the electromagnetic fields of SSP mode will be bounded increasingly to the grooves, yielding a weakly and almost linearly increased effective index (See the supplementary materials):
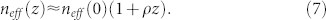
Here ρ is the change rate of effective index (note that ρ is closely proportional to σ, 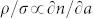, and the quantitative relationship between them can be determined numerically). With this method, the phase velocity *v_p_* = *c*/*n_eff_*(*z*) of the SPP wave will decrease with the length. We anticipate that, with an optimal value of *ρ* or σ, the synchronism interaction between the electron and SSP mode will be prolonged, leading to the enhancement of the interaction efficiency. Because the electron velocity and the phase velocity of SSP mode are not matched completely in the coupling, the above method can thus be termed the quasi-velocity-matching design.

**Figure 3:**
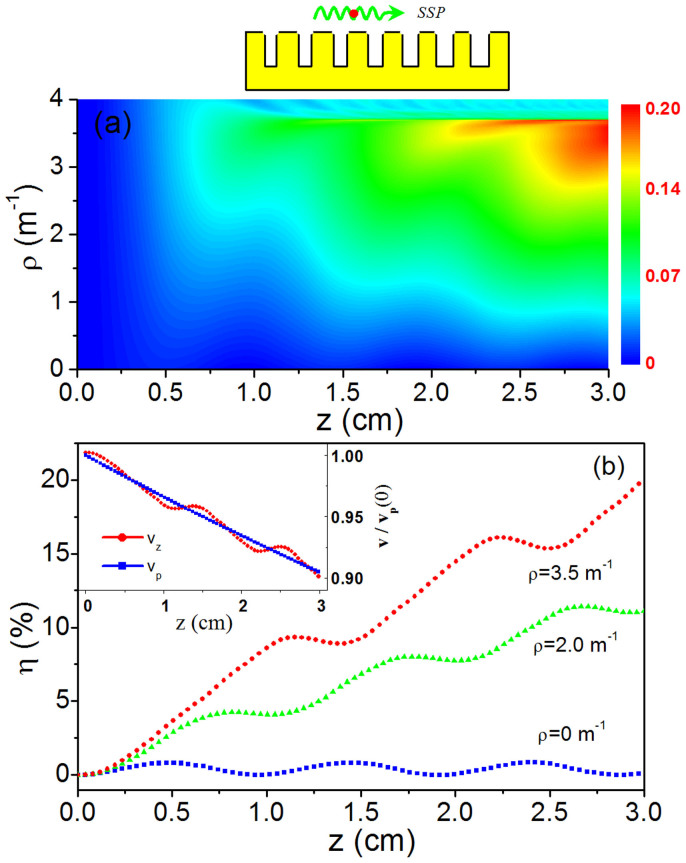
Coupling effect in the plasmonic system with the gradient metasurface or QVM designs. (a) The interaction efficiency as a function of length z and change rate *ρ*. (b) The dependence of efficiency on the length z for *ρ* = 0, 2.0, and 3.5 m^−1^ (the inset shows the evolution of electron velocity v_z_ and phase velocity *v_p_* of SSP with the length, ρ = 3.5 m^−1^). Here, *x*_0_ = 5 *μm*, *v_0_* = 1.002*v_p_*, *B*_0_ = 1*T*, and H_0_ = 0.2 A/mm.

The calculated efficiency based on the QVM design is presented in Fig. 3(a) as a function of length *z* and change rate *ρ*. In the calculation, some modifications have been made in the Eq. 2 due to the varying effective index: 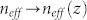, 
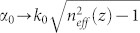
, and 
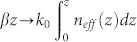
 (the externally-excited SSP mode and other input parameters are the same as the case in Fig. 2(a); *x*_0_ = 5 *μm*). The result is quite different from that in Fig. 2: the original oscillation behavior has been lifted and the interaction efficiency increases almost continuously with the length. When ρ is increased to 3.5 m^−1^, the efficiency reaches 20.0%, which is about 25 times larger than that achieved in Fig. 2 (~0.8%) (The optimal value of ρ depends on the structure parameters as well as the amplitude of SSP mode). But when ρ is further increased (such as 3.7 m^−1^), the efficiency will drop significantly. In this case, the SSP mode is excessively decelerated and velocity matching between the electron and SSP cannot be achieved. As a comparison, Fig. 3(b) presents the efficiency vs. z for *ρ* = 0, 2.0, and 3.5 m^−1^, respectively. The situation is very similar to the quasi- phase-matched frequency conversion in nonlinear optics^23^,^24^. In addition, the inset of Fig. 3(b) presents the evolution of normalized electron velocity (z component, *v_z_*/*v_p0_*) and phase velocity of SSP (*v_p_*/*v_p0_*) with the length (ρ = 3.5 m^−1^). One can see that, in the whole interaction length, the two velocities are close to each other, thus suggesting a good QVM. These results demonstrate that the QVM design based on the gradient effective index is indeed workable.

### Numerical simulations of the coupling effect

**Figure 4:**
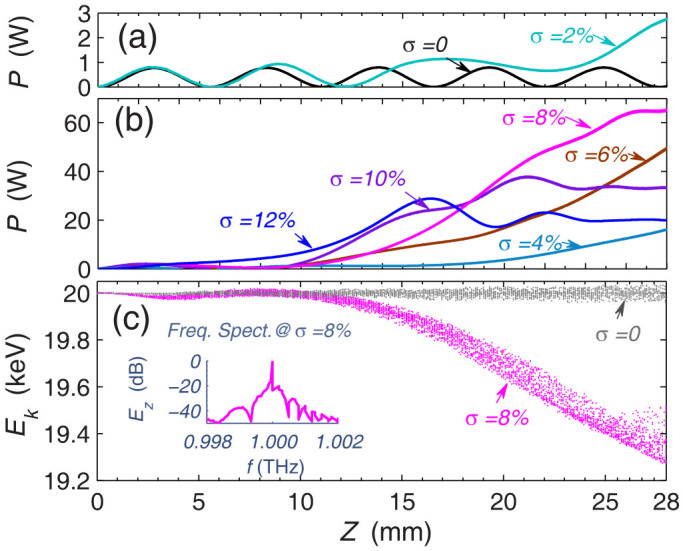
PIC simulations of THz wave generation with the gradient metasurface or QVM designs. (a) and (b) The output power of SSP for various *σ*. (c) The electron beam energy evolution profiles for σ = 0 and 8% (*ρ* = 1.23 *m*^−1^). The inset shows the frequency spectrum of the THz SSP mode for σ = 8%. Here, the SSP mode is self-excited and amplified by the relativistic electrons.

Considering the above results, we further perform the PIC simulations (based on the FDTD method) to test the possible effect^25^. Here, the lattice constant *d*, groove depth *h*, and the guiding magnetic field are the same as that used in Fig. 3. Note that no externally excited SSP mode is employed here; the THz SSP will be self-excited and amplified by the relativistic electrons alone. Figures 4(a) and 4(b) show the simulated output power of the excited THz wave for various change rate *σ* of the groove width (the initial groove width is *a* = 15 μm, and the initial kinetic energy of electron is 20 KeV with *v*_0_ ≈ *c*/3.69). When σ = 0, the power of THz wave oscillates periodically with the length; the maximal power is 0.78 W, corresponding to an efficiency of 0.04% (due to the self-excitation of SSP, this efficiency is lower than that in Fig. 2). But, for the larger value of σ (2%, 4%, 6%, 8%), the output power of SSP grows with the length significantly. For σ = 8% (or *ρ* = 1.23 *m*^−1^), for example, the maximal output power attains 65.2 W at z = 28 mm; the efficiency is 3.26%, two orders of magnitude larger than that achieved with σ = 0. When σ is further increased to 10% and 12%, however, the output power will be degenerated. The qualitative conclusion agrees with that obtained in Fig. 3.

Figure 4(c) presents the energy evolution profile of electron beam during its traveling along the metasurface (where σ = 0 and σ = 8%). It confirms again that with the QVM design, the oscillation behavior of coupling is lifted and the energy of beam electrons can be extracted continuously with the interaction length. The inset in Fig. 4(c) is the frequency spectrum [20log(*E_z_*(*f*)/*E_z_*(1 *THz*)) vs. *f*] of the tangent electric-field component of the excited THz wave (σ = 8%). The result clearly indicates that the system works well in a narrow frequency band with the central frequency locating around 1.0 THz (where the initial ratio of electron velocity to phase velocity of SSP is *v_e_*/*v_p_* ≈ 1.04). In addition, the beam electron trajectories in phase space also suggest that in our system there is no obvious impinging of electrons on the metasurface (See the supplementary materials). These PIC simulations demonstrate that the plasmonic structures and QVM design may provide an efficient tool for the generation and amplification of THz SSP waves.

## Discussions

It should be mentioned that the effect studied here is different from the Smith- Purcell radiation (SPR) effect. It is well known that when a relativistic electron flies along a periodic metal grating surface, the electromagnetic *radiation* modes will be produced due to the interaction between the electron and grating (the peak radiation wavelength is comparable with the grating period)^26^,^27^,^28^. But here, the relativistic electrons couple strongly with the *bounded* electromagnetic surface mode, i.e., the SSP mode confined on the subwavelength gradient metasurfaces (In practice, the length of metasurface is finite. The enhanced SSP wave will be diffracted at the end of metasurface, giving rise to enhanced far-field radiation). Moreover, according to the Ref. 27, the radiation energy of dominant fundamental mode of SPR can be estimated as 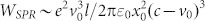, where *l* is the motion distance of electron. Compared with the kinetic energy of relativistic electron, *W_K_* = *m*_0_*c*^2^(*γ*_0_ − 1), the radiation energy of SPR is completely negligible (for *l* = 28 mm, *η_SPR_* = *W_SPR_*/*W_K_* ~ 4 × 10^−5^). With the use of Fabry-Perot resonance amplification, SPR-based devices such as the orotrons may exhibit enhanced radiation energy. But in the THz band, the output power of orotrons is typically of tens of milliwatt. The other vacuum electron devices such as the gyrotrons are bulky in sizes; and, to work in the THz band, a gigantic magnetic field about 10 ~ 20 T is needed. In contrast, our structure is compact, efficient, and it works only with weak guiding magnetic field.

In summary, the strong coupling between the relativistic electrons and SSP mode (which is either excited externally or self-excited by the beam electrons) has been studied. The SSP mode on the subwavelength-structured metasurface can induce both strong tangent electric field and large effective index at the THz band. This provides a strong force for the electron deceleration and a vacuum circumstance for the coupling, where the electron may travel with velocity comparable to or larger than the phase velocity of electromagnetic waves. We suggest theoretically that, by using a weakly gradient metasurface or QVM design, the synchronism interaction between the electron and SSP will be prolonged and the electron energy can be extracted continuously. The PIC simulations also demonstrate that with the QVM design a narrow-band and efficient THz wave can be generated by the relativistic electrons. Compared with the homogeneous metasurfaces, the output power or efficiency is enhanced by two orders of magnitude. These results could be useful for the design of new compact and efficient THz wave sources.

## Methods

The field distribution of externally-excited SSP mode was simulated with the finite- difference time-domain (FDTD) method^18^. In the simulation, the incident light was focused on the narrow air gap (set by the vertical metal screen and the metasurface), generating the SSP mode via diffraction of light. The metal was treated as perfect electric conductor (PEC) and open boundary conditions have been used for the metasurface. To simulate the coupling effect between the relativistic electrons and self-excited SSP mode, a standard Particle-In-Cell (PIC) method used in the study of high power microwave devices has been employed^25^. In this method, the particle beams are considered as a great number of “macro particles” with finite space size. The simulating is executed in time domains by a series of time steps Δt. In every step, the macro particle positions and velocities are counted firstly to get the currents and charge densities on space grid. Then the Maxwell equation is solved with FDTD on the grid to get the electromagnetic fields. Finally, the Lorentz force on the macro particle is calculated with the particle position and velocities, and the macro particles will move Δt time according to the Newton-Lorentz equation. The above steps will be iterated many times and the physical evolution of the beams and electromagnetic field can be traced. In the simulation, the electron beam pulses are injected into the system periodically, where both the beam width and the distance from beam bottom to the metasurface are set as 6 *μm*; the beam current is I = 1 A, the kinetic energy of electron is 20 KeV, the injection period and duration time are 1 ps and 0.1 ps, respectively.

## Author Contributions

L.B.K. and C.P.H. conceived the design. L.B.K., C.P.H. and P.K.L. wrote the main manuscript text. C.H.D. and X.G.Y. performed the numerical simulations.

## Supplementary Material

Supplementary Information Supplementary Information
