# Supplementary material for: Enhancing spoof surface-plasmons with gradient metasurfaces
Source: Sci Rep. 2015 Mar 5;5:8772. doi: 10.1038/srep08772 (PMC4350089; doi:10.1038/srep08772)
Supplement: Supplementary Information [file srep08772-s1.doc]

***Supplementary materials for “Enhancing spoof surface-plasmons with gradient metasurfaces”***

Ling-Bao Kong, Cheng-Ping Huang^*^, Chao-Hai Du, Pu-Kun Liu^*^, and Xiao-Gang Yin

* Email: [cphuang@njtech.edu.cn](mailto:cphuang@njtech.edu.cn) & pkliu@pku.edu.cn

**I. The synchronism condition for the SSP wave amplification**

In this section, we will present the energy exchange mechanism of the electron beam and SSP wave, and the condition of SSP amplification will be discussed.

The SSP wave is a transverse-magnetically polarized surface mode with the *E_x_*, *E_z_*, and *H_y_* components. The *E_x_* component, which is normal to the metal surface, can induce a small oscillation in the *x* direction (see the Sec. IV). The *E_z_* component, which is parallel to the metal surface and the initial electron velocity, will exert a strong force on the electron, thus accelerating or decelerating the electron along the z axis. Since the SSP mode is a slow wave with the phase velocity $v_{p}=c/n_{eff}$, the relativistic electron can travel with velocity *v_e_* comparable to or larger than *v_p._* The interaction process can be divided into three cases:

(1) When the velocity $v_{e}$ of an electron is much larger or smaller than phase velocity $v_{p}$ of the SSP wave, there will be a rapid relative motion of electron with respect to the electromagnetic fields. In this case, the electron moves alternately in the accelerating and decelerating phase of the $E_{\text{z}}$ component, thus dose not lead to any efficient energy exchange between the electron and SSP mode.

(2) Now we consider the case that the velocity of electron and phase velocity of SSP mode are perfectly matched, that is $v_{e}=v_{p}$ (see Fig. S1). When the electron locates at the position 1 or 3, the force acting on the electron is zero. So these electrons will move with the SSP without an interaction. When the electron locates at other positions such as 2 and 4, the situation will be different: a negative force (4) or positive force (2) is resulted, thus decelerating or accelerating the electron. In this case, two important consequences can be seen: one is the breaking of the perfect velocity matching ($v_{e}\neq v_{p}$), and the other is the bunching of electrons near the position 3, as shown in Fig. S2. Thus, about one half of the beam electrons locate at the accelerating phase and the other half at the decelerating phase. Consequently, there is no net energy exchange between the electrons and SSP either.


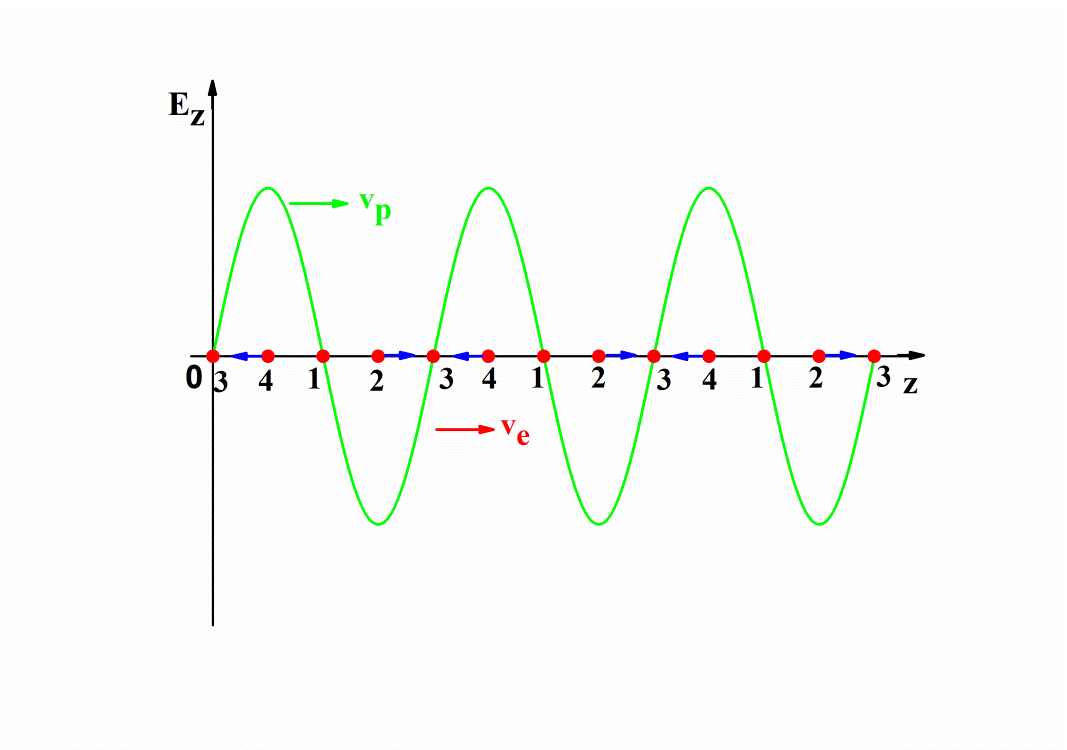


*Fig. S1. The electron motion in the SSP field E_z_.*


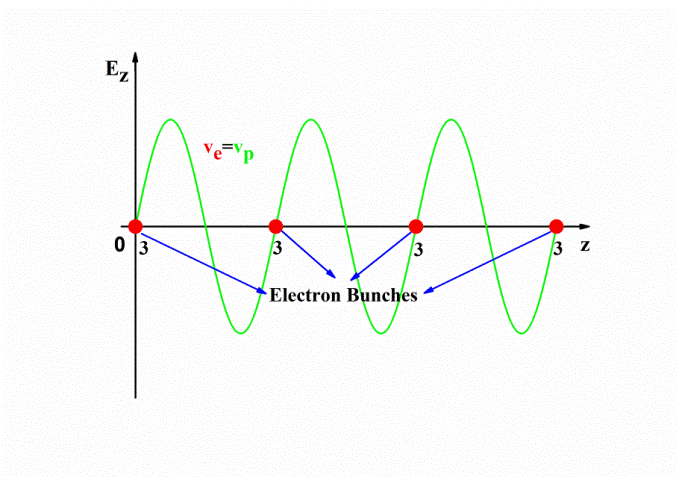


*Fig. S2. The bunching process of beam electrons for* $v_{e}=v_{p}$.

(3) Another situation is that the electron velocity $v_{e}$ is close to but slightly larger than the phase velocity $v_{p}$, where a slow relative motion of electron with respect to the SSP mode is present. In this case, the bunching process that is similar to the case of $v_{e}\text{=}v_{p}$ will also take place, as shown in Fig. S3. But, due to the relative motion of electrons in the fields, the electron bunches move into the decelerating phase and will keep synchronism in decelerating phase for a longer time or traveling distance. Consequently, more energy will be extracted from the beam electrons, leading to the amplification of SSP wave. However, when the electron bunches are decelerated sufficiently and shifted to the accelerating phase, the energy will be transferred back to the electrons again. The alternating deceleration and acceleration of electron bunches give rise to the efficiency oscillation with larger amplitude. In addition, when the electron velocity $v_{e}$ is close to but slightly smaller than the phase velocity $v_{p}$, an inverse coupling process will be resulted.


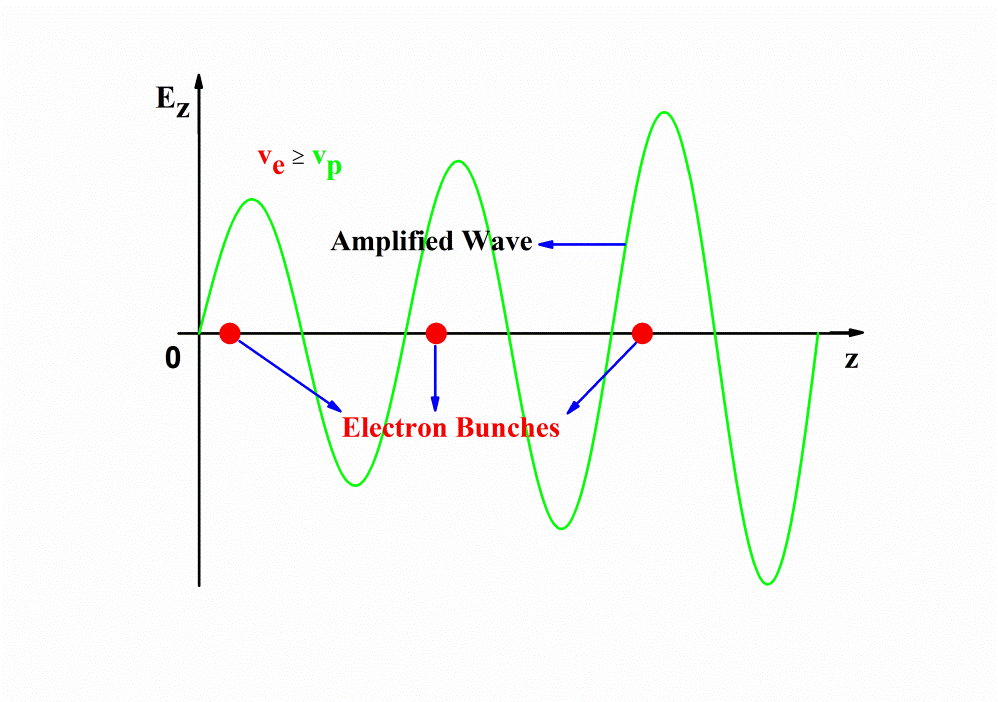


*Fig. S3. The amplification of the SSP wave.*

**II. Effect of initial phase of SSP mode on the coupling**

The initial phase φ of SSP mode is also important for the coupling effect. In our paper, we mainly focus on the case that φ=0. Nonetheless, a nonzero phase φ also works with a high efficiency. The input electron (initially locates at z=0, point 3 of Fig. S1) interacting with SSP of nonzero φ is equivalent to the following case: the electron locating initially at nonzero z (e.g., point 4 & 2 of Fig. S1) interacts with SSP of zero φ. Thus the effect of initial phase of SSP can be analyzed in terms of the initial location z_0_ of electron in the z-axis. When φ is set such that the electron locates around the crest of SSP, a larger decelerating force will act on the electron, yielding a higher efficiency. Figure S4(a) presents the efficiency of single electron at different initial phase of SSP, where the meta-surface is homogeneous (ρ=0). An oscillation of efficiency with the length can be seen. The amplitude of oscillation depends on the phase. When φ is changed from 0 to 45, 90, and 135 degrees, the maximal efficiency increases from 0.83% to 1.8%, 2.8%, and 3.5%, respectively.

To understand the effect, we calculated the relative motion of electron in the z direction with respect to the SSP wave: $\delta z=z_{e}-v_{p}t$. The results are given in Fig. S4(b) as a function of time. For φ=0 (initially the electron is at point 3, see Fig. S1), the electron experiences a positive (or forward) relative motion δz=3 μm (and then goes back to point 3), due to the larger initial velocity of electron and the weak decelerating electric field *E_z_*. For φ=45, 90, and 135 degrees (initially the electron is around the point 4), a stronger *E_z_* is exerted on the electron. Thus, the electron decelerates quickly and then undergoes a significant negative motion (-20 ~ -58 μm). The allowed deceleration length for 135 degrees (~$3\lambda/8n_{eff}=29 \mu m$) is longer than other cases, thus corresponding to a higher coupling efficiency.

*Fig. S4 (a) The efficiency of single electron at various φ. (b) The relative motion of electron with respect to the SPP for various φ. Here ρ=0.*

For the gradient meta-surface, the efficiency of single electron at various initial phases is also calculated, as shown in Fig. S5 (ρ=3.5 *m^-1^*). When φ=0, 45, and 90 degrees, similar results can be seen: the efficiency increases efficiently with the length. The reason is that the phase velocity of SSP mode is also decreased, thus acting on the electron with a continuous decelerating force. Nonetheless, for φ=135 degrees (the initial position of electron is more close to point 1 of Fig. S1), the result is quite different: no continuous increase of efficiency can be obtained. In this case, the electron can pass through the point 1 and go into the accelerating phase. Thus, a continuous deceleration of electron will not occur.

*Fig. S5 The efficiency of single electron at various φ (ρ=3.5 m^-1^).*

For the quasi-continuous injection of electrons (where each electron corresponds to a different position z or equivalently to a different initial phase of SSP mode), a reduced (average) efficiency can still be produced (see Fig. 2b of the paper). In this case, the average efficiency is not sensitive to the initial phase of SSP.

**III. The role of higher-order diffraction modes**

Generally speaking, the high-order modes are also strongly localized with a much lower phase velocity and a much larger attenuation factor, which will contribute little to the electron-wave interactions. When the boundary conditions of the electric and magnetic fields are taken into consideration, the complete set of space harmonics of the SSP excited above the corrugated metal surface can be written as [1]:

$\begin{matrix} H_{y}=\sum_{n} B\frac{\omega\varepsilon_{0}}{\alpha_{n}}\mathrm{sinc}\frac{\beta_{n}a}{2}e^{-\alpha_{n}x}e^{i[\beta_{n}z-\omega t]}, \\ E_{x}=\sum_{n} B\frac{\beta_{n}}{\alpha_{n}}\mathrm{sinc}\frac{\beta_{n}a}{2}e^{-\alpha_{n}x}e^{i[\beta_{n}z-\omega t]}, \\ E_{z}=\sum_{n} \frac{B}{i}\mathrm{sinc}\frac{\beta_{n}a}{2}e^{-\alpha_{n}x}e^{i[\beta_{n}z-\omega t]}. \end{matrix}$ (R1)

Here, *B* is a coefficient determined by the input power of the incident wave, $\alpha_{n}=\sqrt{\beta_{n}^{2}-k_{0}^{2}}$, and $\beta_{n}=\beta+G_{n}=\beta+2\pi n/d$.

Specifically, for the parameters chosen in our paper, we have the effective refractive index of the zeroth- and first-order modes as $n_{eff}=\beta/k_{0}=3.835$ and $n_{eff1}=\beta_{1}/k_{0}=16.335$, respectively. Correspondingly, the phase velocity $v_{p1}$ of the first-order mode is much lower than $v_{p}$ of the zeroth-order one ($v_{p1}\approx0.235v_{p}$). Thus, there is a significant velocity mismatch between the electron velocity $v_{0}=1.002v_{p}$ and the phase velocity $v_{p1}$, and the synchronism condition of the electron to the first-order mode will be destroyed. In addition, the ratio of attenuation factor of the first-order mode to the zeroth-order one is $\alpha_{1}/\alpha_{0}=4.4$, meaning that the first-order mode decays more strongly in the *x* direction. Taking the tangent electric-field as an example, the amplitude ratio of the first-order mode to the zeroth-order one can be determined as $\left|E_{z1}/E_{z0}\right|=(\mathrm{sinc}\frac{\beta_{1}a}{2}/\mathrm{sinc}\frac{\beta a}{2})e^{(\alpha-\alpha_{1})x}$. When *x*=5 μm, the ratio of $E_{z}$ component of the two modes is about 0.06. Thus, for the larger *x*, the contribution of high-order modes is small.

To demonstrate the above conclusion, we theoretically studied the interaction between the electron and the total fields (the other parameters are the same as that used in Fig. 2(a) of the paper). In Fig. S6, the calculated efficiencies for ρ=0 (the homogenous meta-surface) and 3.5 m^-1^ (the gradient meta-surface) are presented as a function of length (see the red solid lines; $x_{0}=5\mu m$). For comparison, the theoretical results for the zeroth-order mode method are also plotted in Fig. S6 (see the blue dash lines). We can see that the efficiencies obtained with the total fields are in good accordance with the zeroth-order approximation.

*Fig. S6. The efficiency of electron interacting with the total fields (solid lines) or zeroth-order mode (dash lines), respectively.*

*Fig. S7. Electron oscillation in the x direction for (a) ρ=0 and (b) 3.5 m^-1^.*

Additionally, the spatial oscillation of electron along the x-axis was mapped in Fig. S7 for ρ=0 and 3.5 m^-1^, respectively. Compared with the Fig. S6, a relatively larger modification of the result is introduced, because of the larger near-field gradient of the higher-order modes. Nonetheless, the characteristics of spatial oscillation can be well captured with the zeroth-order approximation. Based on these results, we can conclude that the fundamental mode is helpful to simplify the analysis and sufficient to elucidate the basic physics of electron-SSP interactions.

**IV. Gradient effective index for the linearly-varying groove width**

The dispersion relation of SSP mode is dependent on the structure parameters such as the groove depth *h* and groove width *a*. The variation of structure parameters in the space will modify the interaction between the surface mode and plasmonic structure, thus altering the mode propagation properties such as the effective index and phase velocity. For example, when the groove width is changed linearly with the length $a\left(z\right)=a\left(1+\sigma z/l\right)$, a gradient effective index may be introduced. We found that if the change rate σ of the groove width is small, the effective index also varies approximately in a linear manner. To illustrate this point, we set *f*=1 THz, *d*=24 μm, *a*=15 μm, *l*=30 mm, and σ=8% (see Fig. 4 of the paper). In this case, the groove width changes slightly from 15 μm to 16.2 μm in a 30 mm-long length. Correspondingly, the effective index changes from 3.835 to 3.976 (the relative variation is 3.68%), as shown in Fig. S8 (the circles). The *n-z* curve has been fitted by using a line $n\left(z\right)=n_{0}\left(1+\rho z\right)$, where *n_0_*=3.837 and ρ=1.23 *m^-^*^1^.

*Fig. S8. Linear variation of groove width and effective index with length.*

**V. The electron oscillations in the x direction**

The SSP mode also presents a strong electric field *E_x_*, which is normal to the metal surface. The oscillating electric field *E_x_* will act on the electrons, thus pulling the electrons to the surface or pushing the electrons away from the surface (this point has been demonstrated by the calculations). This is a serious problem that cannot be avoided in the strong coupling regime. In our system, we use a static magnetic field (along the z axis) to bind the transverse motion of the electrons. In this case, the electrons can be well confined near the structure surface but with a small oscillation in the x direction. Figure S9 presents the calculated results for x_0_=5, 15, and 25 μm (corresponding to Fig. 2(a) of the paper). For $x_{0}=5\mu m$, the electron oscillates with $1.78 \mu m\leq x\leq5.80 \mu m$, thus avoiding the impinging on the structure surface. For the larger x_0_, the oscillating amplitude in the x axis is even smaller, because of the attenuation of SSP from the metal surface. In addition, for the gradient groove widths or QVM design as studied in Fig. 3 of the paper, similar results for the oscillation can be obtained (see Fig. S7b).

*Fig. S9. The oscillation of electrons in the x direction.*

In the self-excitation of THz SSP process (see Fig. 4 of the paper), the injected electron beams will induce and interact with the SSP. Correspondingly, the electron oscillation along the x-axis is also present. The PIC results of the beam electron trajectories in phase space also show that there is no obvious interception of electrons on the structure surface. The details for the case σ=8% are shown in Fig. S10.

*Fig. S10. The beam electron trajectories for σ=8% at t=19.9 ns.*

**Reference:**

[1] Zhang, K. Q. & Li, D. J. *Electromagnetic Theory for Microwaves and Optoelectronics* (Springer, Berlin, 2008).
